# Intravenous immunoglobulin vs plasma exchange in treatment of mechanically ventilated adults with Guillain-Barré syndrome

**DOI:** 10.11604/pamj.2014.18.35.2911

**Published:** 2014-05-09

**Authors:** Boubaker Charra, Abdelhamid Hachimi, Abdellatif Benslama, Said Motaouakkil

**Affiliations:** 1 Medical Intensive Care Unit, Ibn Rochd University Hospital, Casablanca, Morocco; 2 Medical-Surgical Intensive Care Unit, Mohammed VI University Hospital, Faculty of Medicine and Pharmacology, Cadi Ayyad University, Marrakech, Morocco

**Keywords:** Guillain Barré syndrome, intensive care unit, intravenous immunoglobulin, plasma exchange, mechanical ventilation, recovery

## Abstract

**Introduction:**

The aim of the study is to compare efficacy of IvIg versus PE in treatment of mechanically ventilation adults with GBS in intensive care unit.

**Methods:**

It is a prospective, non randomized study, realized in a medical ICU from 2006 to 2010. We included all patients with GBS who required mechanical ventilation (MV). We defined two groups: group 1 (group treated by IvIg: 0.4 g/kg/day for 5 days) and group 2 (group treated by PE: 4 PE during 10-14 days). We collected demographic characteristics, clinical and therapeutic aspects and outcome. Statistical analysis used: The quantitative variables are expressed on mean ± standard derivation and compared by Student test. The statistic analysis has been based on SPSS for windows. P < 0.05 is considered as significant.

**Results:**

Forty-one patients (21 in group 1 and 20 in group 2) were enrolled. The mean age was 37.4 ± 9.2 years, with a masculine predominance (75.4%). Electromyogram in all patients found acute inflammatory demyelinating polyradiculoneuropathy in 80.5% of patients. The mean length of hospitalization was 45.3 ± 9.2 days. The length of hospitalization of the IvIg group is less long than PE group (p = 0.03). The weaning of the MV was more precocious in IvIg group than PE group (p = 0.01). Also, the beginning of motility recuperation was precocious at IvIg group than PE group (p = 0.04).

**Conclusion:**

Our work reveals a meaningful difference for the MV weaning and precocious recovery in IvIg group compared to PE group.

## Introduction

Guillain-Barré syndrome (GBS) is a demyelinating polyradiculoneuropathy with an acute paralysing disorder, typically symmetric and ascending and areflexia. Incidence varies between 0.66 and 1.79 cases per 100 000 persons in general population [1–6]. About pathogenesis, the aetiologies of GBS remain unclear; however, several findings suggest that causes such as an infection of the respiratory or gastrointestinal tract, vaccinations, surgery and pregnancy generate an abnormal immune response which leads to a destruction of myelin sheaths and/or axons [7–9]. The treatment is based on two mainstays: supportive care and immunomodulatory treatment. Supportive care prevents complications such as deep vein thrombosis, digestive bleeding and infections especially and physiotherapy. Both plasma exchange (PE) and intravenous immunoglobulins (IvIg) are the two immunomodulative treatment. Several studies demonstrated that IvIg and PE are efficacious treatment for GBS [10–13]. Our aim is to compare efficacy of IvIg versus PE in treatment of mechanically ventilation adults with GBS in a medical intensive care unit.

## Methods

It is a prospective, monocentric non randomized study, realized in a medical ICU in Ibn Rochd university hospital of Casablanca which is a tertiary referring medical centre, during 5 years. We included all patients with GBS who required mechanical ventilation (MV). The diagnosis was according to clinical criteria [9]. We defined two groups: group 1 (group treated by IvIg: 0.4 g/kg/day during five days) and group 2 (group treated by PE: 4 PE during 10-14 days). The choice of treatment depends on the economic level of the patient and the presence or not of a contraindication to any of the treatments. We recorded data age, sex, origin of the patient, the reason for admission in ICU, results of CSF study, the mean length of hospitalization, duration of ventilation, the onset of motor recovery, complications and specific treatments including plasmapheresis, and IvIg. We also registered the findings of electrophysiological studies. The median interval between onset of neuropathy and performance of the electrophysiological study was 7.5 days. All patients were ventilated using endotracheal mechanical ventilation then tracheotomised within the first week of hospitalization. Patients were intubated if they had SpO_2_ less than 90% in room air requiring increasing FiO_2_, or showed clinical symptoms of CO_2_ retention. When patients were able to trigger spontaneous breathing, they were changed to a pressure-support spontaneous ventilation mode. Pressure support was gradually decreased to 10 cmH_2_O. If secretions were manageable with good airway reflexes, a daily spontaneous breathing trial (SBT) was performed using a T-piece for 12 to 24 hours. Patients were extubated if SBT was successful. SBT was declared successful if there was no increased work of breathing or apnea, symptoms of hypercapnia, tachycardia and if SpO_2_ remained well compared to pre-SBT value. The quantitative variables are expressed on mean ± standard derivation and compared with Student tes. The statistic analysis has been based on SPSS 10.0 for windows. P < 0.05 is considered as significant.

## Results

Between January 2006 and December 2010, 41 patients were enrolled, 21 in group 1 (IvIg group) and 20 in group 2 (PE group). No medical history was found in all patients. The mean age was 37.4 ± 9.2 years, with a masculine predominance (75.4%). There was a statistically insignificant age between the two groups, 35.4 ± 8.4 years for IvIg group versus 39.3 ± 5.2 years for PE group. Symptoms preceding the onset of GBS were fatigue in all patients, gastro-intestinal infections in 13 (32%) patients and nasopharyngitis in 21 (51.2%). The main initial sign was limb weakness followed by muscle pain in all patients and paresthesia in 20 (49%) patients. The mean time from the onset to the maximum of illness in all patients was 8.3 ± 4.2 days. There was no involvement of the cranial nerves in all patients. Autonomic dysfunction was reported in 20 (49%) during hospitalisation such hypotension-hypertension and/or bradycardia and/or excessive sweating. The reason for admission in the ICU was respiratory impairment. Twenty eight patients were admitted from the emergency department, 13 patients were transferred from the department of neurology. Lumbar puncture was performed on all patients; the mean of CSF protein was elevated (0.95 ± 0.1 g/l) in 29% of patients without CSF cell change. Based on electrophysiological findings, in 33 (80.5%) patients had acute inflammatory demyelinating polyradiculoneuropathy (AIDP) and acute motor axonal neuropathy (AMAN) in 8 (19.5%) patients. The mean length of hospitalization was 45.3 ± 9.2 days (range 30 to 118 days). The ICU stay was significantly shorter (p = 0.03) in the IvIg group than PE group. Patients receiving IvIg were early weaned of MV (p = 0.01) compared to those receiving PE with a statistical significance. Also, the beginning of motility recuperation was significantly precocious (p = 0.04) in IvIg group than PE group (Table 1). Both groups had no significant complications due to treatment.

**Table 1:** Comparison of IvIg and PE groups regarding length of stay, beginning of motility recuperation and weaning of mechanical ventilation

	Group 1 (IvIg)	Group 2 (PE)	p
Length of stay in ICU (days)	38.2 ± 7.6	52.4 ± 5.3	0.03
Beginning of motility recuperation (days)	10.43	18.74	0.04
Weaning of mechanical ventilation (days)	18.72	38.52	0.01

**IvIg:** intravenous immunoglobulin; **PE:** plasma exchange

## Discussion

Our results suggest that IvIg is more benefit for our patients than PE. For the IvIg group, the ICU stay was shorter, weaned earlier of MV and the beginning of motility recuperation was precocious than PE group. According to two reviews published in the Cochrane library 2012, patients treated within two weeks from onset with IvIg had recovery as much as PE [14]; and compared to the symptomatic care alone, patients treated by PE had a good evolution [15]. However, some studies suggest that patients had IvIg treatment had more improvement than those had PE. Indeed, Kuwabara [16] and Van der Meché [12] showed that IvIg group had a significant fast evolution than PE group. Contrary, El-Bayoumi et al, in an infant population, found that the PE group had a significant shorter MV duration compared to IvIg group [17]. Finally, no significant difference between the two treatments showed by others authors [18–21]. Furthermore, other therapeutic options are under research such adapted IvIg dosage, complement inhibitors, selective immunoadsorption and FC fragment of IvIg [22].

## Conclusion

Although the results of the literature are not conclusive, our work of which the most important slant is the absence of randomization, reveals that there is a meaningful difference for the MV weaning and a precocious recovery in IvIg group compared to the PE group. These encouraging results would merit to be confirmed by controlled and randomized works.
